# Evaluation of gefitinib efficacy according to body mass index, body surface area, and body weight in patients with EGFR-mutated advanced non-small cell lung cancer

**DOI:** 10.1007/s00280-016-3232-2

**Published:** 2017-02-06

**Authors:** Hisao Imai, Tomohito Kuwako, Kyoichi Kaira, Tomomi Masuda, Yosuke Miura, Kaori Seki, Reiko Sakurai, Mitsuyoshi Utsugi, Kimihiro Shimizu, Noriaki Sunaga, Yoshio Tomizawa, Shinichi Ishihara, Takao Ishizuka, Akira Mogi, Takeshi Hisada, Koichi Minato, Atsushi Takise, Ryusei Saito, Masanobu Yamada

**Affiliations:** 1 Department of Medicine and Molecular Science, Gunma University Graduate School of Medicine, Maebashi, Gunma Japan; 2 Department of Respiratory Medicine, Gunma Prefectural Cancer Center, 617-1, Takahayashi-nishi, Ohta, Gunma 373-8550 Japan; 3 Division of Respiratory Medicine, National Hospital Organization Shibukawa Medical Center, Shibukawa, Gunma Japan; 4 Department of Oncology Clinical Development, Gunma University Graduate School of Medicine, 3-39-15, Showa-machi, Maebashi, Gunma 371-8511 Japan; 5 Division of Internal Medicine, Kiryu Kosei General Hospital, Kiryu, Gunma Japan; 6 Division of General Thoracic Surgery, Integrative Center of General Surgery, Gunma University Hospital, 3-39-15, Showa-machi, Maebashi, Gunma 371-8511 Japan; 7 Division of Internal Medicine, Isesaki Municipal Hospital, Isesaki, Gunma Japan; 8 Division of Internal Medicine, Public Tomioka General Hospital, Tomioka, Gunma Japan; 9 Division of Respiratory Medicine, Maebashi Red Cross Hospital, Maebashi, Gunma Japan

**Keywords:** Advanced non-small cell lung cancer, *EGFR* mutations, Gefitinib, Body surface area, Body weight, Body mass index

## Abstract

**Purpose:**

In patients with epidermal growth factor receptor (*EGFR*)-mutated, advanced, non-small cell lung cancer (NSCLC), common gefitinib-sensitive *EGFR* mutations that predict a greater response to therapy include the exon 19 deletion and L858R point mutation. The objective of this study was to evaluate whether body surface area (BSA), body weight (BW), and body mass index (BMI) affect gefitinib efficacy in such patients.

**Methods:**

The medical charts of 138 consecutive patients with advanced NSCLC harboring sensitive *EGFR* mutations, who underwent gefitinib treatment, were reviewed. The median BSA and BW were used as cutoff values to evaluate their impact on gefitinib efficacy. BMI was categorized as underweight (<18.5 kg/m^2^), normal (18.5–25 kg/m^2^), and overweight (≥25 kg/m^2^).

**Results:**

The median BSA and BW were 1.48 m^2^ and 53 kg, respectively. The overall response rate, progression-free survival (PFS), and overall survival (OS) were 65.2%, 12.2, and 24.2 months, respectively. There were no significant differences in clinical outcomes according to BSA, BW, or BMI alone. Subgroup analysis based on the mutation type and BSA revealed no significant differences in PFS between the groups; however, the median OS in those with exon 19 deletion combined with low BSA was significantly favorable compared with the other groups.

**Conclusions:**

Gefitinib efficacy in patients with NSCLC harboring sensitive *EGFR* mutations did not differ according to BSA, BW, and BMI. However, OS was superior in patients with both the exon 19 deletion and low BSA.

**Electronic supplementary material:**

The online version of this article (doi:10.1007/s00280-016-3232-2) contains supplementary material, which is available to authorized users.

## Introduction

Lung cancer is the most common cause of cancer-related mortality worldwide, with non-small cell lung cancer (NSCLC) accounting for ~85% of all cases [1]. Most patients with NSCLC are diagnosed at the advanced stages (Stages IIIB and IV), which are associated with particularly poor prognoses.

Gefitinib, an epidermal growth factor receptor tyrosine kinase inhibitor (EGFR-TKI), is a first-line treatment for patients with NSCLC harboring sensitive *EGFR* mutations [2–4]. Although many patients initially achieve clinical remission or disease control with first-line chemotherapy, most subsequently experience disease progression and death. The high response rate (RR) for gefitinib is associated with the presence of tumor cells harboring active *EGFR* mutations such as in-frame deletions in exon 19 or point mutations in exon 21 (e.g., L858R) [5–7]. Several phase III trials, which compared platinum-containing chemotherapy to gefitinib in the first-line setting, demonstrated that gefitinib significantly improved progression-free survival (PFS) in patients with *EGFR*-activating mutations [2–4]. Meta-analyses have clearly indicated improved PFS and superior RRs in patients with *EGFR* mutations who underwent EGFR-TKI therapy compared with those who underwent chemotherapy using cytotoxic drugs [8–10]. Therefore, EGFR-TKIs are considered a standard regimen for patients with advanced NSCLC harboring *EGFR* mutations.

The IDEAL 1 and IDEAL 2 studies determined the standard dose of gefitinib to be 250 mg/day, which has been prescribed irrespective of body size [11, 12]. However, this dose was determined before *EGFR* mutations were identified as predictors of PFS and RR to EGFR–TKIs. A previous study demonstrated that body surface area (BSA) influenced gefitinib efficacy in patients with advanced NSCLC who harbored *EGFR* mutations [13, 14], although contrary reports finding no effect of BSA also exist [15]. While BSA-based and body weight (BW)-based dose adjustments are made for chemotherapy with cytotoxic agents and bevacizumab-based chemotherapy, respectively, it is unknown whether BSA- or BW-based dose adjustments could affect gefitinib efficacy in patients with NSCLC harboring *EGFR* mutations. Furthermore, a previous clinical trial reported that higher body mass index (BMI) among patients with advanced NSCLC significantly affected survival [16]. The objective of the present study was to determine whether BSA, BW or BMI could affect the efficacy of first-line gefitinib in Japanese patients with advanced NSCLC harboring gefitinib-sensitive *EGFR* mutations. We also evaluated PFS and overall survival (OS) according to *EGFR* mutation type (exon 19 deletion and exon 21 L858R) because they constitute ~90% of all EGFR mutation-positive lung adenocarcinomas, and are strongly associated with robust responses to EGFR-TKIs [17, 18]. Moreover, patients with EGFR exon 19 deletion tumors have shown improved outcomes with EGFR-TKIs compared to patients with exon 21 L858R-positive tumors [19, 20].

## Materials and methods

In this retrospective study, we evaluated 138 consecutive patients with advanced NSCLC harboring sensitive *EGFR* mutations who were treated with first-line gefitinib, between January 2006 and December 2012, across 7 Japanese institutions (Gunma Prefectural Cancer Center, Gunma University Hospital, National Hospital Organization Shibukawa Medical Center, Kiryu Kosei General Hospital, Isesaki Municipal Hospital, Public Tomioka General Hospital, and Maebashi Red Cross Hospital). The histological diagnosis and staging of NSCLC were based on the World Health Organization classification and the tumor–node–metastasis staging system [21], respectively. The eligibility criteria included patients with histologically or cytologically confirmed advanced NSCLC (unresectable stage III/IV disease or recurrence according to the new Union for International Cancer Control criteria, version 7) whose tumors harbored drug-sensitive *EGFR* mutations (exon 19 deletion/exon 21 L858R), and who were undergoing continuous first-line gefitinib treatment. Patients who did not have at least one measurable lesion according to the Response Evaluation Criteria in Solid Tumors (RECIST) 1.1 criteria [22] were excluded. Before therapy, each patient underwent a physical examination, chest radiography, thorax and abdomen computed tomography, bone scintigraphy or ^18^F-fluorodeoxyglucose positron emission tomography, and brain computed tomography or magnetic resonance imaging to determine the TNM stage. Patient characteristics including sex, age at diagnosis, Eastern Cooperative Oncology Group (ECOG) performance status (PS) at the start of gefitinib treatment, clinical stage, tumor histology, smoking status, *EGFR* mutation type, BW, height, and number of regimens following first-line gefitinib were determined by a retrospective chart review. Patients were classified according to smoking status as current smokers, former light smokers (having smoked for a total of ≤10 pack-years plus smoking cessation at least 15 years previously), and never-smokers (a lifetime history of having smoked <100 cigarettes). We used the following formula to calculate BSA: BSA (m^2^) = [body weight (kg)]^0.425^ × [height (cm)]^0.725^ × 0.007184 [23]. A clinical chart search was performed for each selected patients at each hospital. The Institutional Review Boards of each institution approved the study protocol, although the requirement for written informed consent was waived.

Genomic DNA was extracted from the tumor samples, and EGFR mutations in exons 18–21 were analyzed as previously described [24, 25]. All patients were EGFR-TKI naïve, and underwent first-line oral gefitinib (250 mg/day). Treatment continued until disease progression, the appearance of intolerable toxicity, or withdrawal of consent.

The best overall response and maximum tumor shrinkage were recorded as the tumor responses. Radiographic tumor responses were defined according to the RECIST v1.1 [22] as complete response (CR), the disappearance of all target lesions; partial response (PR), a decrease in the sum of the target lesion diameters of at least 30% compared with baseline; progressive disease (PD), an increase of at least 20% in the sum of the target lesion diameters compared with the smallest sum during the study; stable disease (SD), insufficient shrinkage or expansion to qualify as PR or PD. The differences in the RRs according to the patients’ characteristics were compared using Fisher’s exact test. The distributions of categorical characteristics according to whether the patients’ BSA was ≥1.48 m^2^ (high-BSA group) or <1.48 m^2^ (low-BSA group) were compared using Fisher’s exact test. The distributions of categorical characteristics according to whether patients’ BW was ≥53 kg (high-BW group) or <53 kg (low-BW group) were compared using Fisher’s exact test. BMI at the start of treatment was defined as weight (kg) divided by the height (m) squared. Patients were stratified into BMI groups as defined by the World Health Organization: underweight (BMI < 18.5 kg/m^2^), normal weight (BMI 18.5–25 kg/m^2^), and overweight (BMI ≥ 25 kg/m^2^). PFS was calculated from the start of treatment until PD or death from any cause, and OS was recorded from the first day of treatment until death or was censored at the date of the last follow-up. Survival curves were plotted using the Kaplan–Meier method and the differences in survival times were analyzed using the log-rank test. A *p* value <0.05 was considered statistically significant. After the failure of first-line gefitinib therapy, patients were permitted any subsequent treatment(s), including the continuation of gefitinib treatment. All statistical analyses were performed using JMP, version 11.0, for Windows (SAS Institute, Cary, NC).

## Results

### Patient characteristics

The clinical characteristics of all 138 patients are listed in Table 1. The majority of patients had a good PS (0–1), adenocarcinoma, and stage IV disease or postoperative recurrence. There were some uneven distributions in patient characteristics between the groups evaluated (Table 2). Compared with those in the low-BSA group, in the high-BSA group a greater percentage were male (*p* < 0.0001), were younger than 75 years old (*p* < 0.0007), and were current or former smokers (*p* < 0.0005). Similarly, compared with those in the low-BW group, in the high-BW group a greater percentage of patients were male (*p* < 0.0002), were younger than 75 years old (*p* < 0.017), and were current or former smokers (*p* < 0.003). There were no significant differences in patient characteristics for any of the BMI groups.

**Table 1:** Patient characteristics

Characteristic	*n* (%)
Sex	
Male	36 (26.0)
Female	102 (74.0)
Age (years), median (range)	73 (39–88)
<75	79 (57.2)
≥75	59 (42.8)
Performance status	
0	54 (39.1)
1	54 (39.1)
2	15 (10.8)
3	13 (9.2)
4	2 (1.8)
Clinical stage	
IIIB	10 (7.2)
IV or postoperative recurrence	128 (92.8)
Histology	
Adenocarcinoma	135 (97.8)
Other/not specified	3 (2.2)
Smoking history	
Current or former	36 (26.0)
Never	102 (74.0)
*EGFR* mutation	
Exon 19 deletion	66 (47.9)
Exon 21 L858R	72 (52.1)
BSA (m^2^), median (range)	1.48 (1.09–1.98)
<1.48	69 (50.0)
≥1.48	69 (50.0)
BW (kg), median (range)	53 (32.0–95.6)
<53	67 (48.5)
≥53	71 (51.5)
BMI (kg/m^2^)	
<18.5	23 (17.0)
18.5–25	82 (58.6)
≥25	33 (24.4)

Performance status was determined using the Eastern Cooperative Oncology Group criteria

*EGFR* epidermal growth factor receptor, *BSA* body surface area, *BW* body weight, *BMI* body mass index

**Table 2:** Characteristics according to BSA, BW, and BMI

Characteristic	BSA (m^2^), *n* (%)		p*	BW (kg), *n* (%)		p*	BMI (kg/m^2^), *n* (%)			p*
	≥1.48	<1.48		≥53	<53		<18.5	18.5–25	≥25	
Sex										
Male	29 (80.6)	7 (19.4)	<*0.0001*	28 (77.8)	8 (22.2)	*0.0002*	3 (8.3)	24 (66.7)	9 (25.0)	0.28
Female	40 (39.2)	62 (60.8)		43 (42.2)	59 (57.8)		20 (19.6)	58 (56.9)	24 (23.5)	
Age (years)										
<75	46 (63.9)	26 (36.1)	*0.0007*	44 (61.1)	28 (38.9)	*0.017*	11 (15.3)	43 (59.7)	18 (25.0)	0.88
≥75	23 (34.8)	43 (65.2)		27 (40.9)	39 (59.1)		12 (18.2)	39 (59.1)	15 (22.7)	
PS										
0–1	56 (51.8)	52 (48.2)	0.40	58 (53.7)	50 (46.3)	0.31	14 (12.9)	68 (63.0)	26 (24.1)	0.07
2–4	13 (43.3)	17 (56.7)		13 (51.5)	17 (48.5)		9 (30.0)	14 (46.7)	7 (23.3)	
Clinical stage										
IIIB	4 (40.0)	6 (60.0)	0.51	5 (50.0)	5 (50.0)	0.92	2 (20.0)	6 (60.0)	2 (20.0)	0.93
IV or rec	65 (50.8)	63 (49.2)		66 (51.6)	62 (48.4)		21 (16.4)	76 (59.4)	31 (24.2)	
Smoking history										
Current or former	27 (75.0)	9 (25.0)	*0.0005*	26 (72.2)	10 (27.8)	*0.003*	4 (11.1)	23 (63.9)	9 (25.0)	0.57
Never	42 (41.2)	60 (58.8)		45 (44.1)	57 (55.9)		19 (18.6)	59 (57.9)	24 (23.5)	
*EGFR* mutation										
Exon 19 deletion	34 (51.5)	32 (48.5)	0.73	38 (57.6)	28 (42.4)	0.16	8 (12.1)	44 (66.7)	14 (21.2)	0.21
Exon 21 L858R	35 (48.6)	37 (51.4)		33 (45.8)	39 (54.2)		15 (20.8)	38 (52.8)	19 (26.4)	

Performance status was determined using the Eastern Cooperative Oncology Group criteria

*BSA* body surface area, *BW* body weight, *BMI* body mass index, *PS* performance status, *EGFR* epidermal growth factor receptor

*Fisher’s exact test. Italic *p* values are statistically significant (*p* < 0.05)

### Response to gefitinib according to BSA, BW, and BMI

An objective response was obtained in 90 of 138 patients, including a CR in 11 patients, and the overall RR was 65.2%. The objective tumor responses according to patient characteristics are shown in Supplementary Table 1. The RRs were 63.8 and 66.7% in the high- and low-BSA groups, respectively (*p* = 0.72). The RRs were 63.4 and 67.2% in the high- and low-BW groups, respectively (*p* = 0.64). Furthermore, the RR was 73.9% in the underweight group, 62.2% in the normal weight group, and 66.7% in the overweight group (*p* = 0.56). There were no significant differences in sex, age, PS, clinical stage, smoking history, or type of *EGFR* mutation between the groups.

### PFS after gefitinib therapy according to BSA, BW, and BMI alone

**Fig. 1:**
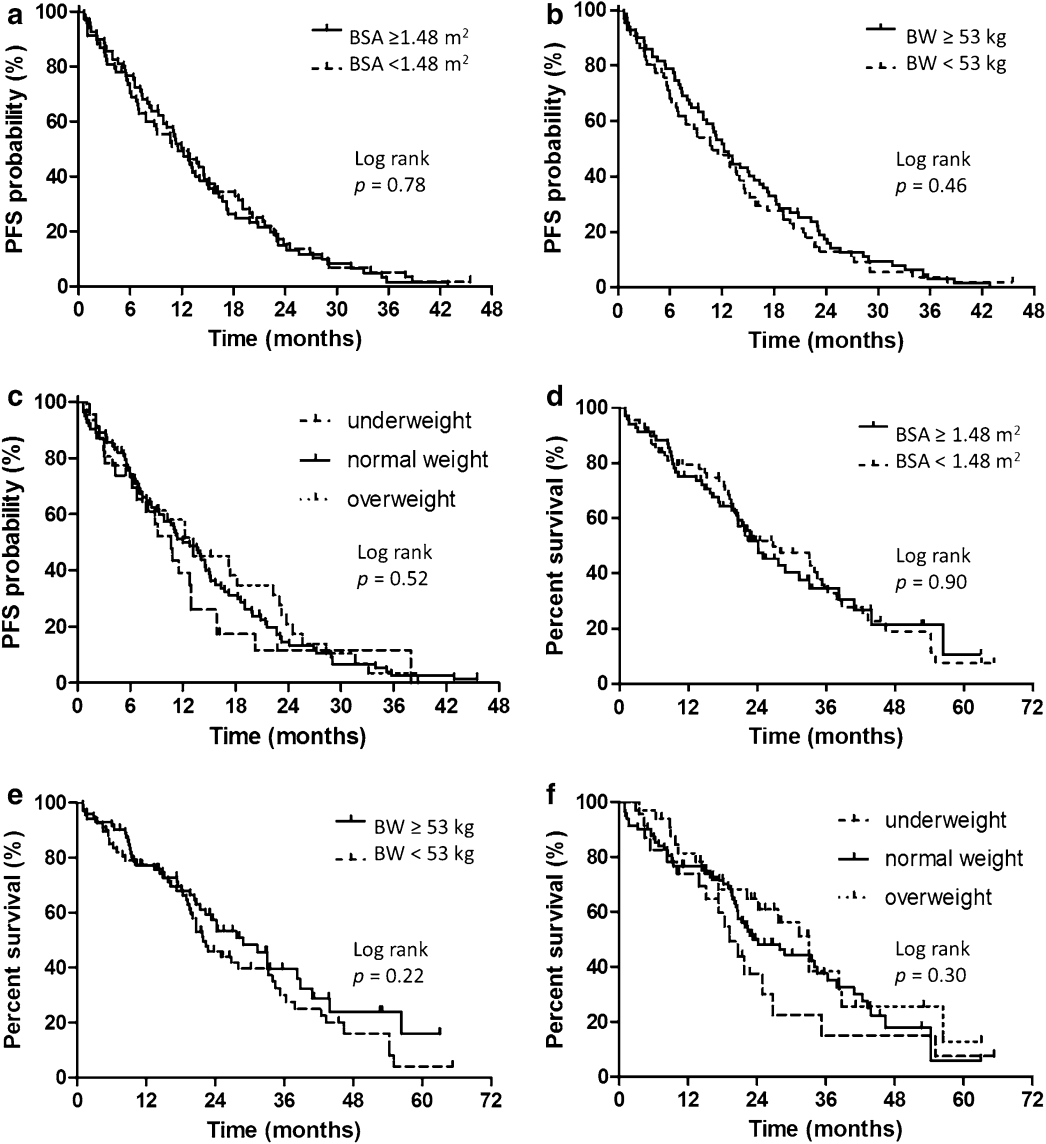
Kaplan–Meier plots showing progression-free and overall survival according to the measured parameters. **a** The median PFS times in the low- and high-BSA groups. **b** The median PFS times in the low- and high-BW groups. **c** The median PFS times in the underweight, normal weight, and overweight groups. **d** The median OS times in the low- and high-BSA groups. **e** The median OS times in the low- and high-BW groups. **f** The median OS times in the underweight, normal weight, and overweight groups, respectively

The median PFS time for the whole study population was 12.0 (95% CI 9.8–14.0) months. The median PFS times in the low- and high-BSA groups were 11.5 (95% CI 7.7–15.1) and 12.2 (95% CI 9.2–14.1) months, respectively (*p* = 0.78, log-rank, Fig. 1a). We also evaluated 1.30, 1.40, 1.50, and 1.60 m^2^ as BSA cutoff values in the log-rank analysis, but none of the differences in PFS were significant (BSA 1.30 m^2^: low vs. high 12.9 vs. 11.3 months, *p* = 0.52; BSA 1.40 m^2^: low vs. high 11.5 vs. 12.2 months, *p* = 0.99; BSA 1.50 m^2^: low vs. high 11.5 vs. 12.2 months, *p* = 0.90; BSA 1.60 m^2^: low vs. high 12.0 vs. 12.2 months, *p* = 0.96).

The median PFS times in the low- and high-BW groups were 10.8 (95% CI 6.9–14.5) and 12.2 (95% CI 9.8–15.6) months, respectively (*p* = 0.46, log-rank, Fig. 1b). We also evaluated 40, and 60 kg as BW cutoff values in the log-rank analysis, but none of the differences in PFS were significant (BW 40 kg: low vs. high 9.1 vs. 12.2 months, *p* = 0.79; BW 60 kg: low vs. high 12.0 vs. 12.2 months, *p* = 0.79).

The median PFS times were 10.6 (95% CI 6.1–12.9), 12.0 (95% CI 9.0–15.1), 13.1 (95% CI 7.0–18.2) months in the underweight, normal weight, and overweight groups, respectively (*p* = 0.52, log-rank, Fig. 1c).

### OS after gefitinib therapy according to BSA, BW, and BMI alone

The median OS time for the whole patient population was 24.2 (95% CI 20.7–33.1) months. The median OS times in the low- and high-BSA groups were 26.8 (95% CI 20.1–35.3) and 24.2 (95% CI 19.6–33.1) months, respectively (*p* = 0.90, log-rank, Fig. 1d).

The median OS times in the low- and high-BW groups were 21.9 (95% CI 19.2–33.2) and 28.9 (95% CI 21.4–38.8) months, respectively (*p* = 0.22, log-rank, Fig. 1e).

The median OS times were 19.2 (95% CI 13.9–26.8), 23.3 (95% CI 20.6–34.5), and 33.1 (95% CI 17.3–38.8) months in the underweight, normal weight, and overweight groups, respectively (*p* = 0.30, log-rank, Fig. 1f).

### PFS and OS to gefitinib therapy according to *EGFR* mutation type and BSA

**Fig. 2:**
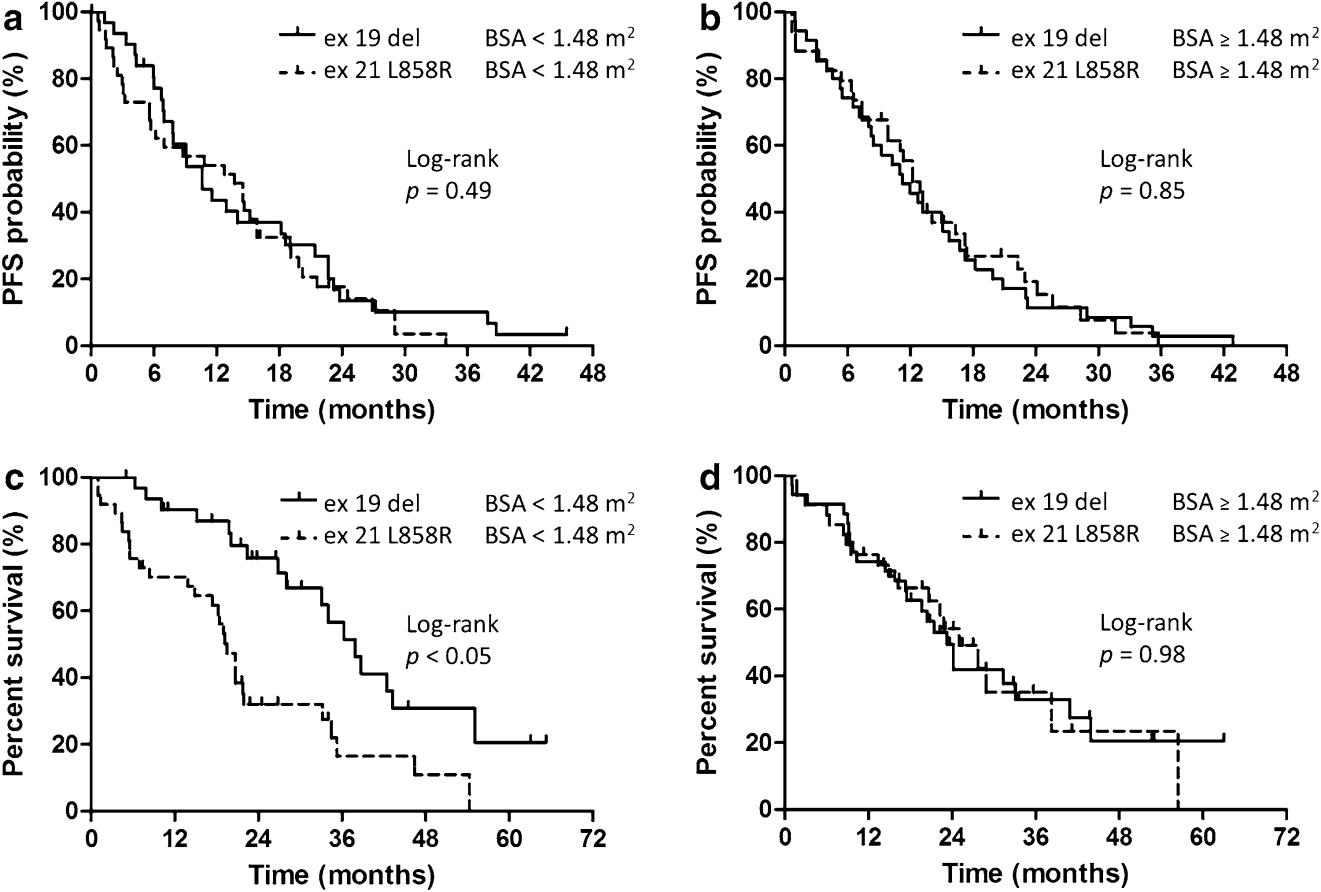
Kaplan–Meier plots showing progression-free and overall survival according to both BSA and mutation type. **a** The median PFS times in the exon 19 deletion_low-BSA and exon 21 L858R_low-BSA groups. **b** The median PFS times in the exon 19 deletion_high-BSA and exon 21 L858R_high-BSA groups. **c** The median OS times in the exon 19 deletion_low-BSA and exon 21 L858R_low-BSA groups; the difference was statistically significant. **d** The median OS times in the exon 19 deletion_high-BSA and exon 21 L858R_high-BSA groups

PFS and OS were analyzed according to *EGFR* mutation type and BSA. The median PFS times were 10.6 (95% CI 6.9–18.6) and 13.7 (95% CI 5.7–15.8) months in the exon 19 deletion_low-BSA and exon 21 L858R_low-BSA groups, respectively (*p* = 0.49, log-rank, Fig. 2a). The median PFS times were 11.2 (95% CI 8.0–15.1) and 12.2 (95% CI 7.4–16.3) months in the exon 19 deletion_high-BSA and exon 21 L858R_high-BSA groups, respectively (*p* = 0.85, log-rank, Fig. 2b).

The median OS times were 37.9 (95% CI 28.0–55.1) and 19.5 (95% CI 13.9–21.9) months in the exon 19 deletion_low-BSA and exon 21 L858R_low-BSA groups, respectively (*p* < 0.05, log-rank, Fig. 2c). The median OS times were 23.3 (95% CI 17.3–40.9) and 25.0 (95% CI 16.2–38.3) months in the exon 19 deletion_high-BSA and exon 21 L858R_high-BSA groups, respectively (*p* = 0.98, log-rank, Fig. 2d).

Therefore, because the median OS times in the exon 19 deletion_low-BSA group were significantly favorable, the predictive value of various clinical factors on PFS and OS for patients with low-BSA was assessed (Table 3). In the univariate analysis, age and PS were significantly associated with superior PFS. In the multivariate analysis, adjusted for various clinical factors, age (p = 0.01) and PS (p = 0.01) were independently associated with superior PFS. Elderly age (≥75 years) was associated with a longer median PFS compared with younger age (<75 years) (14.6 vs. 8.7 months, log-rank *p* = 0.02). Similarly, a better PS score (0–1) was associated with a longer median PFS compared with poorer PS scores (2–4) (14.5 vs. 5.6 months, log-rank *p* < 0.01). In the univariate analysis, PS and *EGFR* mutation type were significantly associated with superior OS. In the multivariate analysis, adjusted for various clinical factors, PS (p < 0.01) and *EGFR* mutation type (p < 0.01) were independently associated with superior OS. Better PS scores (0–1) were associated with a longer median OS compared with worse PS scores (2–4) (33.2 vs. 10.1 months, log-rank *p* < 0.01). Similarly, patients with an exon 19 deletion had a longer median OS compared to those with an exon 21 L858R mutation (37.9 vs. 19.5 months, log-rank, *p* < 0.01).

**Table 3:** Associations between clinical factors, and progression-free survival and overall survival for patients with low body surface area (<1.48 m^2^)

Factors	Univariate analysis PFS			Multivariate analysis PFS			Univariate analysis OS			Multivariate analysis OS		
	HR	95% CI	*p* value	HR	95% CI	*p* value	HR	95% CI	*p* value	HR	95% CI	*p* value
Sex (male/female)	1.63	0.62–3.54	0.29	2.18	0.80–5.08	0.11	1.68	0.49–4.31	0.36	1.73	0.49–4.72	0.35
Age (<75/≥75 years)	1.76	1.05–2.93	*0.03*	2.03	1.12–3.67	*0.01*	0.74	0.39–1.33	0.32	0.79	0.39–1.55	0.50
PS (0–1/2–4)	0.42	0.24–0.79	<*0.01*	0.41	0.24–0.85	*0.01*	0.28	0.14–0.58	<*0.01*	0.26	0.12–0.56	<*0.01*
Clinical stage (III/IV + recurrence)	0.47	0.17–1.04	0.06	0.55	0.20–1.30	0.19	0.50	0.15–1.27	0.16	0.87	0.24–2.36	0.80
*EGFR* type (exon 19 del/exon 21 L858R)	0.83	0.50–1.38	0.49	0.71	0.39–1.26	0.25	0.36	0.19–0.66	<*0.01*	0.41	0.20–0.79	<*0.01*

*BSA* body surface area, *HR* hazard ratio, *CI* confidence interval, *PS* performance status, *EGFR* epidermal growth factor receptor, *PFS* progression-free survival, *OS* overall survival

Italic *p* values are statistically significant (*p* < 0.05)

## Discussion

The present study showed that BSA, BW, and BMI alone did not affect the clinical outcomes of gefitinib therapy including RR, PFS, and OS in patients with advanced NSCLC harboring an *EGFR* mutation. Furthermore, BSA in combination with *EGFR* mutation type did not affect OS. However, in patients with a low BSA (<1.48 m^2^), *EGFR* mutation type significantly affected OS following gefitinib therapy. Specifically, in patients with a low BSA, compared with the L858R mutation, the exon 19 deletion conferred superior OS times following gefitinib therapy.

For the 138 patients in the present study, and those enrolled in 2 Japanese randomized phase III studies (NEJ002 and WJTOG3405) [3, 4], there were no major differences in the distribution of patient characteristics. Therefore, the population in the present study was considered suitable for an evaluation of gefitinib efficacy in patients with advanced NSCLC harboring an *EGFR* mutation.

Ichihara et al. reported a retrospective study showing that BSA affected PFS in patients with NSCLC harboring an EGFR mutation who underwent gefitinib therapy [13]. They stated that a post hoc sub-analysis of the data reported by Ando et al. supported their findings, i.e., that BSA values ≥1.5 m^2^ were associated with an inferior response to gefitinib [26]. However, Ando et al. performed their analysis irrespective of EGFR mutation status. A previous study found that EGFR mutations were more frequent among non-smokers than smokers, and more frequent among females than males [27]. Therefore, it is reasonable to assume that in Ando et al., there were more male patients with wild-type EGFR compared to females with an EGFR mutation in the high-BSA group, and, accordingly, the high-BSA group had poor responses to gefitinib in their study [26]. In fact, Ando et al. found that BSA was not independently predictive of antitumor response survival [26].

In the present study, subgroup analyses examining different *EGFR* mutations in the low-BSA group showed a significant improvement in OS in patients with tumors harboring the exon 19 deletion mutation compared with that in patients with tumors harboring the exon 21 L858R mutation. By contrast, there were no significant differences in OS in the high-BSA group according to the type of mutation present. As previously mentioned, these 2 mutations constitute the majority of *EGFR* mutation-positive lung adenocarcinomas [17] and can affect responses and outcomes to EGFR-TKIs [18–20, 28]. However, the reasons for such differences in response to EGFR-TKIs are unknown. One possibility is that exon 19 deletion harboring tumors are more efficiently inhibited by gefitinib compared with exon 21 L858R mutation harboring tumors, although in vitro studies do not support this hypothesis. NSCLC cell lines bearing exon 19 deletion or L858R mutations showed similar growth inhibition profiles in response to gefitinib or erlotinib [29, 30]. Furthermore, EGFR phosphorylation is completely inhibited by equivalent concentrations of gefitinib in NIH-3T3 cells expressing either an exon 21 L858R mutation or an exon 19 deletion [30]. An alternative hypothesis is that the exon 20 T790M mutation, which has been associated with acquired resistance to EGFR-TKIs [31, 32], might occur more frequently in the presence of an exon 21 L858R mutation compared with in the presence of an exon 19 deletion. However, the presently available data are too limited to draw any such conclusions, and continued analyses of tumor specimens from patients who develop acquired resistance to gefitinib or erlotinib are needed to answer this question.

Although mutation type affected the median OS in the low-BSA group, it did not affect the median PFS. This is most likely because of bias in the number of patients between the two groups. However, one possibility is that patients with exon 19 deletion-positive tumors have improved outcomes following EGFR-TKIs therapy compared to those with exon 21 L858R mutations, as mentioned above. To date, dose adjustment according to BSA has not been approved for any molecular-targeted agent. Although erlotinib and afatinib require such analysis, our findings suggest that the potential influence of BSA on the pharmacokinetic and pharmacodynamic variability of EGFR-TKIs could be considered the next step in investigating the clinical meaningfulness of BSA-based dosing during EGFR-TKI treatment.

This study has several limitations. First, it was a retrospective analysis, and the results could not be regarded as completely definitive. Second, reducing, skipping, or delaying the planned chemotherapy was performed at the attending physician’s discretion. To minimize potential bias, all consecutive patients who were treated at our institutions were included in the analysis, and the patients’ original charts were thoroughly reviewed. Third, the study sample size was small, although we believe that the results of the present study are useful because it includes a greater number of patients compared with any previous reports. Fourth, we did not include pharmacokinetic data in the analysis; therefore, it is unclear whether BSA differences led to inter-patient pharmacokinetic variability, resulting in the observed differences in OS. A pharmacokinetics–pharmacodynamics study is warranted to clarify this issue. Finally, since the cutoff value for BSA was different from that commonly used in the Western countries (i.e., 1.7 m^2^) [33], further assessments would be needed to extrapolate the findings to Europeans and North Americans. Because of these limitations, the data should be interpreted with caution.

In conclusion, the efficacy of gefitinib in patients with advanced NSCLC harboring sensitive *EGFR* mutations does not differ according to BSA, BW, or BMI. However, the OS of those with an exon 19 deletion combined with low BSA was significantly favorable. Our findings suggest that adjustment of gefitinib dose based on *EGFR* mutation type could be considered the next step in investigating the clinical implications of BSA-based dosing during gefitinib monotherapy.

## Electronic supplementary material

Below is the link to the electronic supplementary material.

Supplementary material 1 (DOCX 16 KB)

## References

[1] DeSantis CE, Lin CC, Mariotto AB, Siegel RL, Stein KD, Kramer JL, Alteri R, Robbins AS, Jemal A (2014). Cancer treatment and survivorship statistics, 2014. CA Cancer J Clin.

[2] Mok TS, Wu YL, Thongprasert S, Yang CH, Chu DT, Saijo N, Sunpaweravong P, Han B, Margono B, Ichinose Y, Nishiwaki Y, Ohe Y, Yang JJ, Chewaskulyong B, Jiang H, Duffield EL, Watkins CL, Armour AA, Fukuoka M (2009). Gefitinib or carboplatin-paclitaxel in pulmonary adenocarcinoma. N Engl J Med.

[3] Mitsudomi T, Morita S, Yatabe Y, Negoro S, Okamoto I, Tsurutani J, Seto T, Satouchi M, Tada H, Hirashima T, Asami K, Katakami N, Takada M, Yoshioka H, Shibata K, Kudoh S, Shimizu E, Saito H, Toyooka S, Nakagawa K, Fukuoka M, West Japan Oncology Group (2010). Gefitinib versus cisplatin plus docetaxel in patients with non-small-cell lung cancer harbouring mutations of the epidermal growth factor receptor (WJTOG3405): an open label, randomised phase 3 trial. Lancet Oncol.

[4] Maemondo M, Inoue A, Kobayashi K, Sugawara S, Oizumi S, Isobe H, Gemma A, Harada M, Yoshizawa H, Kinoshita I, Fujita Y, Okinaga S, Hirano H, Yoshimori K, Harada T, Ogura T, Ando M, Miyazawa H, Tanaka T, Saijo Y, Hagiwara K, Morita S, Nukiwa T, North-East Japan Study Group (2010). Gefitinib or chemotherapy for non-small-cell lung cancer with mutated EGFR. N Engl J Med.

[5] Inoue A, Kobayashi K, Usui K, Maemondo M, Okinaga S, Mikami I, Ando M, Yamazaki K, Saijo Y, Gemma A, Miyazawa H, Tanaka T, Ikebuchi K, Nukiwa T, Morita S, Hagiwara K, North East Japan Gefitinib Study Group (2009). First-line gefitinib for patients with advanced non-small-cell lung cancer harboring epidermal growth factor receptor mutations without indication for chemotherapy. J Clin Oncol.

[6] Morita S, Okamoto I, Kobayashi K, Yamazaki K, Asahina H, Inoue A, Hagiwara K, Sunaga N, Yanagitani N, Hida T, Yoshida K, Hirashima T, Yasumoto K, Sugio K, Mitsudomi T, Fukuoka M, Nukiwa T (2009). Combined survival analysis of prospective clinical trials of gefitinib for non-small cell lung cancer with EGFR mutations. Clin Cancer Res.

[7] Han JY, Park K, Kim SW, Lee DH, Kim HY, Kim HT, Ahn MJ, Yun T, Ahn JS, Suh C, Lee JS, Yoon SJ, Han JH, Lee JW, Jo SJ, Lee JS (2012). First-SIGNAL: first-line single-agent iressa versus gemcitabine and cisplatin trial in never-smokers with adenocarcinoma of the lung. J Clin Oncol.

[8] Bria E, Milella M, Cuppone F, Novello S, Ceribelli A, Vaccaro V, Sperduti I, Gelibter A, Scagliotti GV, Cognetti F, Giannarelli D (2011). Outcome of advanced NSCLC patients harboring sensitizing EGFR mutations randomized to EGFR tyrosine kinase inhibitors or chemotherapy as first-line treatment: a meta-analysis. Ann Oncol.

[9] Petrelli F, Borgonovo K, Cabiddu M, Barni S (2012). Efficacy of EGFR tyrosine kinase inhibitors in patients with EGFR-mutated non-small-cell lung cancer: a meta-analysis of 13 randomized trials. Clin Lung Cancer.

[10] Gao G, Ren S, Li A, Xu J, Xu Q, Su C, Guo J, Deng Q, Zhou C (2012). Epidermal growth factor receptor-tyrosine kinase inhibitor therapy is effective as first-line treatment of advanced non-small-cell lung cancer with mutated EGFR: a meta-analysis from six phase III randomized controlled trials. Int J Cancer.

[11] Fukuoka M, Yano S, Giaccone G, Tamura T, Nakagawa K, Douillard JY, Nishiwaki Y, Vansteenkiste J, Kudoh S, Rischin D, Eek R, Horai T, Noda K, Takata I, Smit E, Averbuch S, Macleod A, Feyereislova A, Dong RP, Baselga J (2003). Multi-institutional randomized phase II trial of gefitinib for previously treated patients with advanced non-small-cell lung cancer (The IDEAL 1 Trial) [corrected]. J Clin Oncol.

[12] Kris MG, Natale RB, Herbst RS, Lynch TJ, Prager D, Belani CP, Schiller JH, Kelly K, Spiridonidis H, Sandler A, Albain KS, Cella D, Wolf MK, Averbuch SD, Ochs JJ, Kay AC (2003). Efficacy of gefitinib, an inhibitor of the epidermal growth factor receptor tyrosine kinase, in symptomatic patients with non-small cell lung cancer: a randomized trial. JAMA.

[13] Ichihara E, Hotta K, Takigawa N, Kudo K, Kato Y, Honda Y, Hayakawa H, Minami D, Sato A, Tabata M, Tanimoto M, Kiura K (2013). Impact of physical size on gefitinib efficacy in patients with non-small cell lung cancer harboring EGFR mutations. Lung Cancer.

[14] Kudo K, Hotta K, Ichihara E, Yoshioka H, Kunimasa K, Tsubouchi K, Iwasaku M, Kato Y, Oze I, Takigawa N, Tanimoto M, Kiura K (2015). Impact of body surface area on survival in EGFR-mutant non-small cell lung cancer patients treated with gefitinib monotherapy: observational study of the Okayama Lung Cancer Study Group 0703. Cancer Chemother Pharmacol.

[15] Igawa S, Kasajima M, Ishihara M, Kimura M, Hiyoshi Y, Niwa H, Kusuhara S, Harada S, Asakuma M, Otani S, Katono K, Sasaki J, Masuda N (2014). Evaluation of gefitinib efficacy according to body surface area in patients with non-small cell lung cancer harboring an EGFR mutation. Cancer Chemother Pharmacol.

[16] Dahlberg SE, Schiller JH, Bonomi PB, Sandler AB, Brahmer JR, Ramalingam SS, Johnson DH (2013). Body mass index and its association with clinical outcomes for advanced non-small-cell lung cancer patients enrolled on Eastern Cooperative Oncology Group clinical trials. J Thorac Oncol.

[17] Rosell R, Moran T, Queralt C, Porta R, Cardenal F, Camps C, Majem M, Lopez-Vivanco G, Isla D, Provencio M, Insa A, Massuti B, Gonzalez-Larriba JL, Paz-Ares L, Bover I, Garcia-Campelo R, Moreno MA, Catot S, Rolfo C, Reguart N, Palmero R, Sánchez JM, Bastus R, Mayo C, Bertran-Alamillo J, Molina MA, Sanchez JJ, Taron M, Spanish Lung Cancer Group (2009). Screening for epidermal growth factor receptor mutations in lung cancer. N Engl J Med.

[18] Sharma SV, Bell DW, Settleman J, Haber DA (2007). Epidermal growth factor receptor mutations in lung cancer. Nat Rev Cancer.

[19] Jackman DM, Yeap BY, Sequist LV, Lindeman N, Holmes AJ, Joshi VA, Bell DW, Huberman MS, Halmos B, Rabin MS, Haber DA, Lynch TJ, Meyerson M, Johnson BE, Jänne PA (2006). Exon 19 deletion mutations of epidermal growth factor receptor are associated with prolonged survival in non-small cell lung cancer patients treated with gefitinib or erlotinib. Clin Cancer Res.

[20] Riely GJ, Pao W, Pham D, Li AR, Rizvi N, Venkatraman ES, Zakowski MF, Kris MG, Ladanyi M, Miller VA (2006). Clinical course of patients with non-small cell lung cancer and epidermal growth factor receptor exon 19 and exon 21 mutations treated with gefitinib or erlotinib. Clin Cancer Res.

[21] Goldstraw P, Crowley J, Chansky K, Giroux DJ, Groome PA, Rami-Porta R, Postmus PE, Rusch V, Sobin L, International Association for the Study of Lung Cancer International Staging Committee; Participating Institutions (2007). The IASLC Lung Cancer Staging Project: proposals for the revision of the TNM stage groupings in the forthcoming (seventh) edition of the TNM Classification of malignant tumours. J Thorac Oncol.

[22] Eisenhauer EA, Therasse P, Bogaerts J, Schwartz LH, Sargent D, Ford R, Dancey J, Arbuck S, Gwyther S, Mooney M, Rubinstein L, Shankar L, Dodd L, Kaplan R, Lacombe D, Verweij J (2009). New response evaluation criteria in solid tumours: revised RECIST guideline (version 1.1). Eur J Cancer.

[23] Du Bois D, Du Bois EF (1989). A formula to estimate the approximate surface area if height and weight be known. 1916. Nutrition.

[24] Nagai Y, Miyazawa H, Huqun, Tanaka T, Udagawa K, Kato M, Fukuyama S, Yokote A, Kobayashi K, Kanazawa M, Hagiwara K (2005). Genetic heterogeneity of the epidermal growth factor receptor in non-small cell lung cancer cell lines revealed by a rapid and sensitive detection system, the peptide nucleic acid-locked nucleic acid PCR clamp. Cancer Res.

[25] Yatabe Y, Hida T, Horio Y, Kosaka T, Takahashi T, Mitsudomi T (2006). A rapid, sensitive assay to detect EGFR mutation in small biopsy specimens from lung cancer. J Mol Diagn.

[26] Ando M, Okamoto I, Yamamoto N, Takeda K, Tamura K, Seto T, Ariyoshi Y, Fukuoka M (2006). Predictive factors for interstitial lung disease, antitumor response, and survival in non-small-cell lung cancer patients treated with gefitinib. J Clin Oncol.

[27] Mitsudomi T, Yatabe Y (2007). Mutations of the epidermal growth factor receptor gene and related genes as determinants of epidermal growth factor receptor tyrosine kinase inhibitors sensitivity in lung cancer. Cancer Sci.

[28] Jackman DM, Miller VA, Cioffredi LA, Yeap BY, Janne PA, Riely GJ, Ruiz MG, Giaccone G, Sequist LV, Johnson BE (2009). Impact of epidermal growth factor receptor and KRAS mutations on clinical outcomes in previously untreated non-small cell lung cancer patients: results of an online tumor registry of clinical trials. Clin Cancer Res.

[29] Paez JG, Janne PA, Lee JC, Tracy S, Greulich H, Gabriel S, Herman P, Kaye FJ, Lindeman N, Boggon TJ, Naoki K, Sasaki H, Fujii Y, Eck MJ, Sellers WR, Johnson BE, Meyerson M (2004). EGFR mutations in lung cancer: correlation with clinical response to gefitinib therapy. Science.

[30] Mukohara T, Engelman JA, Hanna NH, Yeap BY, Kobayashi S, Lindeman N, Halmos B, Pearlberg J, Tsuchihashi Z, Cantley LC, Tenen DG, Johnson BE, Jänne PA (2005). Differential effects of gefitinib and cetuximab on non-small-cell lung cancers bearing epidermal growth factor receptor mutations. J Natl Cancer Inst.

[31] Kobayashi S, Boggon TJ, Dayaram T, Janne PA, Kocher O, Meyerson M, Johnson BE, Eck MJ, Tenen DG, Halmos B (2005). EGFR mutation and resistance of non-small-cell lung cancer to gefitinib. N Engl J Med.

[32] Pao W, Miller VA, Politi KA, Riely GJ, Somwar R, Zakowski MF, Kris MG, Varmus H (2005). Acquired resistance of lung adenocarcinomas to gefitinib or erlotinib is associated with a second mutation in the EGFR kinase domain. PLoS Med.

[33] Sacco JJ, Botten J, Macbeth F, Bagust A, Clark P (2010). The average body surface area of adult cancer patients in the UK: a multicentre retrospective study. PLoS One.

