# Supplementary material for: Evaluation of gefitinib efficacy according to body mass index, body surface area, and body weight in patients with EGFR-mutated advanced non-small cell lung cancer
Source: Cancer Chemother Pharmacol. 2017 Feb 6;79(3):497–505. doi: 10.1007/s00280-016-3232-2 (PMC5344961; doi:10.1007/s00280-016-3232-2)
Supplement: Supplementary file 1 — Supplementary material 1 (DOCX 16 KB) [file 280_2016_3232_MOESM1_ESM.docx]

Supplementary Table 1. Responses according to patient characteristics

| Characteristic |  | Number of responders | Response rate (%) | *p** |
| --- | --- | --- | --- | --- |
| BSA (m^2^) | ≥ 1.48 | 44 | 63.8 | 0.72 |
|  | < 1.48 | 46 | 66.7 |  |
| BW (kg) | ≥ 53 | 45 | 63.4 | 0.64 |
|  | < 53 | 45 | 67.2 |  |
| BMI (kg/m^2^) | < 18.5 | 17 | 73.9 | 0.56 |
|  | 18.5–25 | 51 | 62.2 |  |
|  | ≥ 25 | 22 | 66.7 |  |
| Sex | Male | 24 | 66.7 | 0.83 |
|  | Female | 66 | 64.7 |  |
| Age (years) | < 75 | 50 | 69.4 | 0.27 |
|  | ≥ 75 | 40 | 60.6 |  |
| PS | 0–1 | 73 | 67.6 | 0.26 |
|  | 2–4 | 17 | 56.7 |  |
| Clinical stage | IIIB | 5 | 50.0 | 0.29 |
|  | IV or rec | 85 | 66.4 |  |
| Smoking history | Current or former | 26 | 72.2 | 0.30 |
|  | Never | 64 | 62.6 |  |
| *EGFR* mutation | Exon 19 deletion | 42 | 63.6 | 0.70 |
|  | Exon 21 L858R | 48 | 66.7 |  |

*Fisher’s exact test

Performance status was determined using the Eastern Cooperative Oncology Group criteria. BSA, body surface area; BW, body weight; BMI, body mass index; PS, performance status; rec, recurrence; EGFR, epidermal growth factor receptor
